# The high degree of similarity in histopathological and clinical characteristics between radiogenic and sporadic papillary thyroid microcarcinomas in young patients

**DOI:** 10.3389/fendo.2022.970682

**Published:** 2022-08-19

**Authors:** Tetiana Bogdanova, Serhii Chernyshov, Liudmyla Zurnadzhy, Tatiana I. Rogounovitch, Norisato Mitsutake, Mykola Tronko, Masahiro Ito, Michael Bolgov, Sergii Masiuk, Shunichi Yamashita, Vladimir A. Saenko

**Affiliations:** ^1^ Laboratory of Morphology of Endocrine System, State Institution “VP Komisarenko Institute of Endocrinology and Metabolism of the National Academy of Medical Sciences of Ukraine”, Kyiv, Ukraine; ^2^ Department of Radiation Molecular Epidemiology, Atomic Bomb Disease Institute, Nagasaki University, Nagasaki, Japan; ^3^ Department of Surgery of Endocrine Glands, State Institution “VP Komisarenko Institute of Endocrinology and Metabolism of the National Academy of Medical Sciences of Ukraine”, Kyiv, Ukraine; ^4^ Department of Radiation Medical Sciences, Atomic Bomb Disease Institute, Nagasaki University, Nagasaki, Japan; ^5^ Department of Fundamental and Applied Problems of Endocrinology, State Institution “VP Komisarenko Institute of Endocrinology and Metabolism of the National Academy of Medical Sciences of Ukraine”, Kyiv, Ukraine; ^6^ Department of Diagnostic Pathology, National Hospital Organization Nagasaki Medical Center, Omura, Japan; ^7^ Radiation Protection Laboratory, State Institution “National Research Center of Radiation Medicine of the National Academy of Medical Science of Ukraine”, Kyiv, Ukraine; ^8^ Fukushima Medical University, Fukushima, Japan; ^9^ National Institute of Radiological Sciences, National Institutes for Quantum Science and Technology, Chiba, Japan

**Keywords:** radiogenic papillary thyroid microcarcinoma, sporadic papillary thyroid microcarcinoma, invasiveness, treatment, *BRAF^V600E^* mutation, Ki67 labeling index

## Abstract

The potential overtreatment of patients with papillary thyroid microcarcinoma (MPTC) has been an important clinical problem in endocrine oncology over the past decade. At the same time, current clinical guidelines tend to consider prior radiation exposure as a contraindication to less extensive surgery, even for low-risk thyroid carcinomas, which primarily include microcarcinomas. This study aims to determine whether there are differences in the behavior of MPTC of two etiological forms (radiogenic and sporadic), including invasive properties, clinical data, and recurrence in patients aged up to 30 years. For this purpose, 136 radiogenic (from patients aged up to 18 years at the time of the Chornobyl accident) and 83 sporadic (from patients born after the Chornobyl accident) MPTCs were selected and compared using univariate and multivariate statistical methods in a whole group and in age and tumor size subgroups. No evidence of more aggressive clinical and histopathological behavior of radiogenic MPTCs as compared to sporadic tumors for basic structural, invasive characteristics, treatment options, and postoperative follow-up results was found. Moreover, radiogenic MPTCs were characterized by the lower frequencies of oncocytic changes (OR = 0.392, p = 0.004), nodal disease (OR = 0.509, p = 0.050), and more frequent complete remission (excellent response) after radioiodine therapy (OR = 9.174, p = 0.008). These results strongly suggest that internal irradiation does not affect tumor phenotype, does not associate with more pronounced invasive properties, and does not worsen prognosis in pediatric or young adult patients with MPTC, implying that radiation history may be not a pivotal factor for determining treatment strategy in such patients.

## Introduction

Epidemiological studies have shown a worldwide increase in thyroid cancer incidence in recent decades (1, 2). To a large extent, the increase is due to a more frequent detection of papillary thyroid carcinoma as compared to other thyroid cancer histotypes, and particularly of low-grade tumors (3–8) which intrinsically include papillary thyroid microcarcinomas (MPTCs) measuring up to 10 mm. The increase in the detection of small tumors is attributed to rapid strides in ultrasound diagnostic capabilities, improved fine-needle aspiration biopsy (FNA), introduction of screenings, and increased patient awareness about the availability of facile thyroid examination (3–5, 7).

The notions of “overdiagnosis” and “overtreatment” are actively discussed over the past decade in many areas of contemporary medicine, including management of small thyroid nodules (3–5, 9–11). Avoiding overtreatment of patients with MPTCs became a challenging clinical problem in the field of endocrine oncology. Prospects of changing treatment strategies from total thyroidectomy to organ-preserving surgery or even active surveillance instead of surgery are widely debated in the medical and scientific communities (12–20).

At the same time, clinical guidelines recommend considering prior head and neck radiation of a patient even with low-risk thyroid cancer as a potential contraindication to less extensive surgery, at least in adults (21, 22). Indeed, data obtained in previous years showed histopathological differences between the radiogenic (patients exposed to Chornobyl fallouts in childhood) and sporadic PTCs (non-exposed patients born after the Chornobyl accident) pointing at more aggressive behavior of radiogenic tumors especially in children and adolescents (23–26). Even so, whether this holds true for MPTCs in young patients exposed to radiation remains unclear since previous studies did not specifically address this question and included tumors of all sizes. The current study retrospectively investigates the differences between MPTCs of radiogenic and sporadic etiology in terms of tumor structure and invasiveness, treatment options and follow-up results in young patients aged up to 30 years treated at a single institution.

## Materials and methods

### Patients

The radiogenic series included MPTCs from 136 patients aged 8.8-30.9 years at diagnosis operated on at the State Institution “VP Komisarenko Institute of Endocrinology and Metabolism of the National Academy of Medical Sciences of Ukraine” (IEM), Kyiv during the period from 1992 to 2016 when a significant increase in thyroid cancer incidence after the Chornobyl accident was documented (23, 27, 28). Given that the high thyroid cancer risk was observed in individuals who were children and adolescents at the time of the Chornobyl accident and lived in the six northern, most contaminated by ^131^I regions of Ukraine (23, 27–29), we defined radiogenic cases as those diagnosed in subjects aged up to 18 years in April 1986, who lived in Kyiv, Chernihiv, Zhytomyr, Rivne, Cherkasy regions, and Kyiv city in 1986. For additional analyses, patients were divided into the pediatric (≤18), and young adult (19-30 years old at diagnosis) subgroups.

The sporadic MPTCs were from 83 patients aged 9.7-30.9 years at diagnosis who were born after the Chornobyl accident (from January 1^st^, 1987 or later, i.e. not affected by ^131^I) and lived in the same six northern regions at the time of the operation. Patients were operated on from 2001 to 2018.

Inclusion criteria for both radiogenic and sporadic MPTCs were non-incidental tumor finding (to ensure the adequacy of clinical option comparisons), and the absence of screening history in tumor detection (as screening may be expected to introduce a bias toward smaller and less advanced tumors). Since the oldest patient with sporadic MPTC at the time of this study was aged 30 years, the upper limit for the age of patients with radiogenic MPTC was also set to 30 years old. None of MPTCs in the study was familial.

The study was conducted according to the guidelines of the Declaration of Helsinki and was approved by the IEM Bioethics Committee (protocols N 22-KE of April 26, 2018, and N 31-KE of February 27, 2020), the Chornobyl Tissue Bank (CTB, project N001-2020), and the Ethics Committee of Nagasaki University (protocol 20130401–7 of July 1, 2021, the latest update). Informed consent was obtained from all patients enrolled in the study or their guardians (for minors).

### Histopathology

Histopathological examination of hematoxylin/eosin-stained paraffin sections was performed by two experienced pathologists at IEM (ТB and LZ). The pathological diagnosis was based on the 4^th^ edition of the WHO histological classification (30). Most cases were also reviewed by the international pathology panel of the CTB project (31, 32). The diagnosis of MPTC was confirmed in all analyzed cases. TNM categories were determined according to the 8^th^ edition of the pTNM classification (33). Tumors were also classified according to the dominant histological growth pattern into three categories: papillary, follicular, or solid-trabecular when the corresponding structural component exceeded 50% of a tumor section surface, and also evaluated for the presence of oncocytic (oxyphilic/Hurtle) cell changes in the tumor epithelium.

As in our previous works (26, 34, 35), in addition to conventional clinical-pathological features, we used an integrative variable, the “invasiveness score”, which is the arithmetic sum of every instance of multifocality, lymphatic/vascular invasion, any extrathyroidal extension (i.e., minimal to the fat or connective tissues, or extending to the muscle), pN1 and M1 (commonly detected by diagnostic imaging), either isolated or in combination with other(s), for each tumor. Thus defined, the invasiveness score ranged from 0 (no invasive feature presents) to 5 (all features present). The actual highest invasiveness score observed in the present MPTC series was 4.

### Immunohistochemistry

Immunohistochemical (IHC) staining for BRAF^V600E^ and Ki67 were performed in 34 and 32 radiogenic, and in 38 and 36 sporadic MPTCs, respectively, for which the additional tissue sections were available.

IHC staining for BRAF^V600E^ was performed as described before (34, 35). In brief, a mouse monoclonal anti-BRAF (mutated V600E) antibody (VE1) ab228461(Abcam) at a 1:100 dilution and the Novolink Polymer Detection System (250T) (Leica RE7140-K) were used to detect IHC reaction product. In our hands, the BRAF^V600E^ positivity on IHC was concordant with the presence of the *BRAF^V600E^* mutation (36).

The proliferative activity of tumors was evaluated by IHC using a Ki67 antibody (clone MIB-1; DAKO, Glostrup, Denmark, 1:100 dilution) in a Ventana BenchMark ULTRA instrument. The Ki67 labeling index (Ki67 LI) was determined with the image-analyzing software (CountσCell, Ki67 antigen Semi-Auto Counter, Seiko Tec LTD, Fukuoka, Japan) in a total of approximately 1,000 PTC cells per case (LZ). Image analysis was performed in a blind for the BRAF^V600E^ status manner.

### Thyroid dosimetry

^131^I thyroid radiation doses were calculated for each patient from the radiogenic group in the Radiation Protection Laboratory of the State Institution “National Research Center for Radiation Medicine of the National Academy of Medical Sciences of Ukraine”, Kyiv using an ecological dosimetric model, which includes the system of ecological iodine transport and biokinetic models of iodine (“TD-CTB”) (29). The median (and IQR) ^131^I thyroid radiation dose for radiogenic MPTCs were 106 (56–203) mGy.

### Statistical analysis

The Fisher’s exact test, Fisher-Freeman-Halton exact test, Cochran-Armitage test and the Kaplan-Meier test were used for univariate analysis of categorical data; the Mann-Whitney test was used to compare continuous data between any two groups. Logistic regression models were adjusted for age at operation and sex; models with very small numbers of outcomes (< 5 per cell) were conducted using Firth’s approach to bias-reducing penalized maximum likelihood fit. Multivariable linear regression models were used for continuous dependent variables. The Cox proportional hazard model was used to analyze disease-free survival. Calculations were performed using IBM SPSS Statistics Version 24 software (International Business Machines Corp., Armonk, NY, USA) and/or the 9.4 version of SAS (SAS Institute Inc., Cary, NC, USA). Correspondence analysis was performed in R with the “ca” package (37). The “colgreen” option was used to calculate biplot principal coordinates for the MPTC subgroups and contribution coordinates (the standard coordinates multiplied by the square root of the corresponding masses) for categorical clinicopathological features. All tests were two-sided; p < 0.05 was considered statistically significant. To identify the variables potentially contributing to the differences between the radiogenic and sporadic MPTCs, the adaptive lasso, best subset (maximum 20 variables), pruned forward selection and adaptive elastic net algorithms of variable selection with holdback validation method (0.3 holdback proportion) were applied using the binomial generalized regression in JMP Pro 16.2.0 (SAS Institute Inc., Cary, NC, USA). The non-zeroed variables were considered as potentially relevant. The Akaike information criterion (AIC), and areas under curve (AUC) for training and validation subsets were calculated for each algorithm result.

## Results

### Whole group analysis

**Figure 1:**
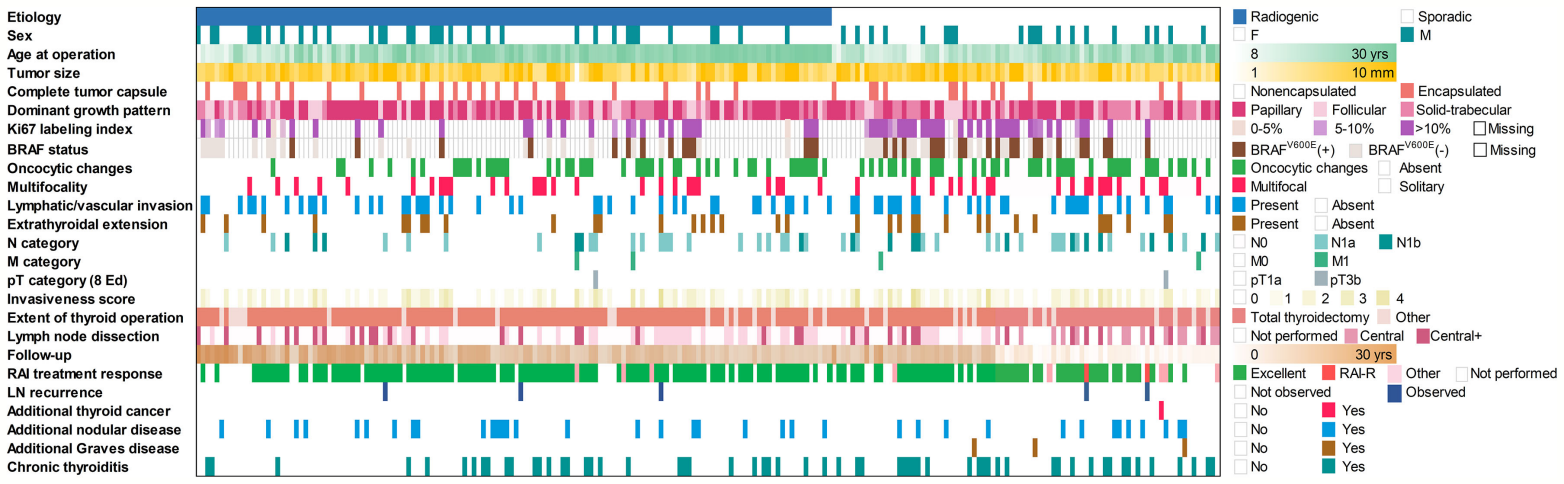
Baseline and clinicopathological profile of 136 radiogenic and 83 sporadic papillary microcarcinomas in the study.

MPTCs detected by ultrasound and assessed by FNA/cytology as malignant or suspicious for malignancy accounted for 136 and 83 cases in the radiogenic and sporadic groups, respectively. All data collected for or generated during this study are presented in **Figure 1**.

Comparison of MPTCs from two etiological groups revealed significantly older age of patients in the radiogenic series (b = 26.519, p = 7.41E-08). Therefore, multivariate analyses were adjusted for this parameter and sex (**Table 1**). Statistically significant differences between the two groups for tumor size, architecture, frequency of the *BRAF^V600E^* mutation or proliferative activity were not observed. The radiogenic MPTCs were less likely to display oncocytic changes (OR = 0.392, p = 0.004), lymph node metastases (OR = 0.509, p = 0.050) and signs of concomitant chronic thyroiditis (OR = 0.431, p = 0.015). Of importance, the response to radioiodine therapy (RIT) was overall better among the radiogenic MPTCs (OR = 9.059, p = 0.008) with the higher frequency of excellent response (i.e., a complete remission, OR = 9.174, p = 0.008) and suggestively lower frequency (in fact, the absence) of radioiodine-refractory (RAI-R) recurrence (OR = 0.090, p = 0.092). Of note, among the recurrent metastases (5 in total), there were 0/3 RAI-R metastases in the radiogenic group and 2/2 in the sporadic group (OR = 0.024, p = 0.389; the lack of statistical significance is likely due to an extremely small number of recurrences).

**Table 1:** Characteristics of the radiogenic and sporadic papillary thyroid microcarcinomas in patients aged up to 30 years.

Parameters	RAD, n = 136	SPOR, n=83	p-value	OR, b or HR (95%CI)	p-value
	number or value (% or IQR)	number or value (% or IQR)	univariate	multivariate^a^	
**Sex** (F/M; %M; F:M ratio, ref=F)	102/34; 25.0%; 3.0:1	63/20; 24.1%; 3.2:1	1.000	1.479 (0.742-2.945)^b^	0.266
**Age at operation**, years	26.4 (22.5-28.8)	21.7 (17.3-24.5)	**1.27E-07**	**26.519 (17.140-35.897)** ^c^	**7.41E-08**
**Tumor size**, mm	7 (6-9)	7 (6-9)	0.893	0.169 (-0.904-1.242)	0.757
1 - 5 mm	22 (16.2%)	17 (20.5%)	0.468	0.628 (0.294-1.343)	0.220
6 - 10 mm	114 (83.8%)	66 (79.5%)	0.468	1.593 (0.745-3.407)	0.230
**Complete tumor capsule**	25 (18.4%)	12 (14.5%)	0.578	1.589 (0.771-3.570)	0.256
**Dominant growth pattern**			0.334	0.858 (0.496-1.485)	0.583
papillary	71 (52.2%)	38 (45.8%)	0.404	1.060 (0.589-1.906)	0.846
follicular	32 (23.5%)	17 (20.5%)	0.621	1.307 (0.644-2.654)	0.458
solid-trabecular	33 (24.3%)	28 (33.7%)	0.162	0.739 (0.386-1.418)	0.364
**Ki-67 labeling index**, median	n = 32; 5.7 (2.9-7.9)	n = 36; 5.0 (3.3-7.8)	0.936	-0.043 (-0.528-0.442)	0.859
0 - 5%	15 (46.9%)	20 (55.6%)	0.627	0.711 (0.239-2.113)	0.539
>5 - 10%	13 (40.6%)	10 (27.8%)	0.311	1.593 (0.506-5.014)	0.426
>10%	4 (12.5%)	6 (16.7%)	0.739	0.829 (0.191-3.604)	0.803
**BRAF^V600E^-positive**	n = 34; 14 (41.2%)	n = 38; 20 (52.6%)	0.355	0.352 (0.115-1.079)	0.068
**Oncocytic changes**	46 (33.8%)	39 (47.0%)	0.063	**0.392 (0.207-0.741)**	**0.004**
**Multifocality**	27 (19.9%)	20 (24.1%)	0.499	0.585 (0.285-1.199)	0.143
**Lymphatic/vascular invasion**	40 (29.4%)	29 (34.9%)	0.454	0.862 (0.463-1.605)	0.640
**Extrathyroidal extension** (any)	19 (14.0%)	16 (19.3%)	0.343	0.872 (0.400-1.899)	0.729
**N category (N1)**	26 (19.1%)	26 (31.3%)	**0.049**	**0.509 (0.259-1.000)**	**0.050**
N1a	20 (14.7%)	15 (18.1%)	0.570	0.684 (0.312-1.500)	0.343
N1b	6 (4.4%)	11 (13.3%)	**0.034**	0.366 (0.124-1.082)	0.069
**M category (M1)**	2 (1.5%)	2 (2.4%)	0.635	0.581 (0.073-4.623)	0.608
**pT**					
pT1a	135 (99.3%)	82 (98.8%)	1.000	1.812 (0.098-33.661)	0.690
pT3b	1 (0.7%)	1 (1.2%)	1.000	0.615 (0.075-5.013)	0.650
**Invasiveness score**	1 (0-1)	1 (0-2)	0.051	0.649 (0.380-1.107)	0.113
0	63 (46.3%)	31 (37.3%)	0.208	1.405 (0.793-2.492)	0.244
1	41 (30.1%)	26 (31.3%)	0.881	0.875 (0.477-1.602)	0.664
2	24 (17.6%)	15 (18.1%)	1.000	0.091 (0.531-2.242)	0.813
3	7 (5.1%)	7 (8.4%)	0.397	0.480 (0.152-1.521)	0.212
4	1 (0.7%)	4 (4.8%)	0.070	0.317 (0.053-1.904)	0.209
**Thyroid surgery volume**					
total thyroidectomy	118 (86.8%)	71 (85.5%)	0.841	1.298 (0.555-3.033)	0.548
organ-preserving operation	18 (13.2%)	12 (14.5%)	0.841	0.771 (0.330-1.801)	0.771
**LN dissection performed**	59 (43.4%)	39 (47.0%)	0.675	0.778 (0.432-1.400)	0.402
level ≥ 6	38 (27.9%)	26 (31.3%)	0.674	0.640 (0.332-1.232)	0.182
level 1 – 5	21 (15.4%)	13 (15.7%)	1.000	1.177 (0.528-2.623)	0.690
**RIT performed**	104 (76.5%)	59 (71.1%)	0.426	1.177 (0.608-2.279)	0.629
**RIT cycles**	1 (1-1)	1 (0-1)	0.155	1.455 (0.818-2.586)	0.202
**Cumulative RI activity,** MBq	3815 (2405-4360)	4360 (3528-4413)	**0.016**	-0.316 (-0.638-0.006)	0.054
**RIT response**	n = 104	n = 59	0.056	**9.059 (1.796-45.698)**	**0.008**
RAI-R recurrence *vs* other	0	2 (3.4)	0.130	0.090 (0.006-1.478)	0.092
excellent *vs* other	101 (97.1%)	52 (88.1%)	**0.037**	**9.174 (1.795-46.876)**	**0.008**
**Follow-up**, years	8.2 (3.2-14.2)	3.8 (1.9-7.6)	**5.47E-06**	**1.793 (1.299-2.287)**	**1.33E-11**
**LN recurrence (after 6 months)**	3 (2.2%)	2 (2.4%)	0.838^d^	0.625 (0.113-3.969)^e^	0.617
**Time to recurrence**, **yrs**	1.2 (1.2-1.2)	1.1 (0.9-1.3)	1.000	0.222 (-3.603-4.047)	0.596
**Recurrent metastases**	n = 3	n = 2			
Dominant growth pattern			1.000	NA^f^	0.668
papillary	2 (66.7%)	1 (50%)	1.000	1.155 (0.002-567.739)	0.964
follicular	1 (33.3%)	0	1.000	16.740 (0.011-inf)	0.450
solid-trabecular	0	1 (50%)	0.400	0.240 (0.000-117.849)	0.652
Ki67 labeling index	n=1; 0.8	n=2; 2.8 (2.5-3.1)	0.221	NA	NA
Oncocytic changes	1 (33.3%)	2 (100%)	0.400	0.031 (0.000-36.002)	0.335
Cystic changes	2 (66.7%)	1 (50%)	1.000	72.239 (0.006-inf^g^)	0.376
RAI-R recurrence *vs* other	0	2 (100%)	0.100	0.024 (0.000-113.649)	0.389
excellent *vs* other	3 (100%)	0	0.100	40.996 (0.009-inf)	0.389
**Additional thyroid cancer**	0	1 (1.2%)	0.379	0.083 (0.005-1.443)	0.088
**Additional nodular disease**	24 (17.6%)	13 (15.7%)	0.853	1.114 (0.503-2.466)	0.790
**Additional Graves disease**	0	3 (3.6%)	0.053	0.065 (0.004-1.051)	0.054
**Chronic thyroiditis**	30 (22.1%)	29 (34.9%)	**0.042**	**0.431 (0.218-0.851)**	**0.015**

a Adjusted for age at operation and sex unless otherwise specified; characteristics of sporadic MPTCs were used as references.

b Adjusted for age at operation.

c Adjusted for sex.

d The log-rank test.

e The Firth’s penalized proportional hazard model.

^f^Not available.

^g^Infinity.

The numbers in bold indicate statistical significance.

Another parameter that was different between the groups was the longer duration of postoperative follow-up (b = 1.793, p = 1.33E-11) of radiogenic MPTCs. It is explained by the earlier calendar time of inclusion of patients with radiogenic MPTCs in the study.

### Pediatric and young adult subgroups

In the pediatric subgroup aged ≤18years at diagnosis (**Table 2**), there were no structural or invasive differences between the radiogenic and sporadic tumors. Only the lower frequencies of oncocytic changes (OR = 0.188, p = 0.034), and lower cumulative ^131^I activity during RIT (b = -0.722, p = 0.021) were observed in the radiogenic subgroup. As in the whole series, the period of postoperative follow-up was longer for the radiogenic MPTCs (b = 2.464, p = 2.00E-06); recurrences were not documented in either etiological subgroup.

**Table 2:** Characteristics of the radiogenic and sporadic papillary thyroid microcarcinomas in pediatric patients aged ≤ 18 years.

Parameters	RAD, n = 23	SPOR, n = 29	p-value	OR, b or HR (95% CI)	p-value
	number or value (% or IQR)	number or value (% or IQR)	univariate	multivariate^a^	
**Sex** (F/M; %M; F:M ratio, ref=F)	12/11; 47.8%; 1.1:1	21/8; 27.6%; 2.6:1	0.157	2.468 (0.763-7.984)^b^	0.132
**Age at operation**, years	15.2 (14.6-17.7)	16.4 (14.0-17.4)	0.978	-0.907 (-8.578-6.765)^c^	0.813
**Tumor size**, mm	7 (6-8)	8 (7-9)	0.134	-1.444 (-3.419-0.531)	0.148
1 - 5 mm	3 (13.0%)	5 (17.2%)	1.000	0.633 (0.127-3.156)	0.577
6 - 10 mm	20 (87.0%)	24 (82.8%)	1.000	1.579 (0.317-7.865)	0.577
**Complete tumor capsule**	4 (17.4%)	5 (17.2%)	1.000	1.050 (0.240-4.599)	0.949
**Dominant growth pattern**			0.879	1.402 (0.473-4.155)	0.542
papillary	8 (34.8%)	11 (37.9%)	1.000	0.683 (0.200-2.335)	0.543
follicular	6 (26.1%)	6 (20.7%)	0.746	1.157 (0.302-4.432)	0.831
solid-trabecular	9 (39.1%)	12 (41.4%)	1.000	1.341 (0.383-4.696)	0.647
**Ki-67 labeling index**, median	n = 12; 3.6 (1.9-6.5)	n = 18; 4.8 (3.5-10.6)	0.094	-0.649 (-1.436-0.138)	0.102
0 - 5%	8 (66.7%)	10 (55.6%)	0.709	1.080 (0.199-5.859)	0.929
>5 - 10%	4 (33.3%)	3 (16.7%)	0.392	4.229 (0.606-29.501)	0.146
>10%	0	5 (27.8%)	0.066	0.106 (0.005-2.243)	0.149
**BRAF^V600E^-positive**	n = 13; 2 (15.4%)	n = 18; 8 (44.4%)	0.129	0.287 (0.046-1.792)	0.182
**Oncocytic changes**	2 (8.7%)	12 (41.4%)	**0.011**	**0.188 (0.040-0.880)**	**0.034**
**Multifocality**	1 (4.3%)	3 (10.3%)	0.621	0.552 (0.080-3.835)	0.548
**Lymphatic/vascular invasion**	9 (39.1%)	11 (37.9%)	1.000	1.261 (0.388-4.101)	0.699
**Extrathyroidal extension (any)**	4 (17.4%)	8 (27.6%)	0.513	0.655 (0.173-2.487)	0.534
**N category (N1)**	4 (17.4%)	7 (24.1%)	0.735	0.596 (0.148-2.401)	0.467
N1a	2 (8.7%)	3 (10.3%)	1.000	0.732 (0.128-4.175)	0.725
N1b	2 (8.7%)	4 (13.8%)	0.682	0.635 (0.114-3.547)	0.604
**M category (M1)**	0	1 (3.4%)	1.000	0.516 (0.032-8.379)	0.641
**pT**					
pT1a	27 (100%)	29 (100%)	NA^d^	NA	NA
pT3b	0	0	NA	NA	NA
**Invasiveness score**	0 (0-1.5)	1 (0-1)	0.412	0.644 (0.227-1.827)	0.408
0	12 (52.2%)	10 (34.5%)	0.262	1.994 (0.627-6.342)	0.242
1	5 (21.7%)	12 (41.4%)	0.152	0.381 (0.104-1.393)	0.145
2	5 (21.7%)	4 (13.8%)	0.486	1.975 (0.472-8.259)	0.351
3	1 (4.3%)	2 (6.9%)	1.000	0.718 (0.090-5.739)	0.755
4	0	1 (3.4%)	1.000	0.433 (0.016-11.827)	0.620
**Thyroid surgery volume**					
total thyroidectomy	19 (82.6%)	27 (93.1%)	0.387	0.322 (0.051-2.018)	0.226
organ-preserving operation	4 (17.4%)	2 (6.9%)	0.387	3.105 (0.496-19.447)	0.226
**LN dissection**	6 (26.1%)	12 (41.4%)	0.379	0.449 (0.129-1.558)	0.207
level ≥ 6	2 (8.7%)	7 (24.1%)	0.268	0.324 (0.064-1.633)	0.172
level 1 – 5	4 (17.4%)	5 (17.2%)	1.000	1.009 (0.207-4.932)	0.991
**RIT performed**	13 (56.5%)	22 (75.9%)	0.234	0.419 (0.121-1.445)	0.169
**RIT cycles**	1 (0-1)	1 (1-1)	0.236	0.388 (0.128-1.174)	0.094
**Cumulative RI activity,** MBq	2405 (2183-2775)	4223 (2390-4436)	0.087	**-0.722 (-1.328- -0.115)**	**0.021**
**RIT response**	n = 13	n = 22			
RAI-R recurrence *vs* other	0	0	NA	NA	NA
excellent *vs* other	13 (100%)	21 (95.5%)	1.000	1.848 (0.096-35.456)	0.684
**Follow-up**, years	19.4 (11.0-21.0)	6.3 (3.4-10.0)	**7.24E-06**	**2.464 (1.553-3.374)**	**2.00E-06**
**LN recurrence after 6 months**	0	0	NA	NA	NA
**Additional thyroid cancer**	0	0	NA	NA	NA
**Additional nodular disease**	3 (13.0%)	5 (17.2%)	1.000	0.803 (0.165-3.909)	0.786
**Additional Graves disease**	0	1 (3.4%)	1.000	0.350 (0.020-6.063)	0.470
**Chronic thyroiditis**	2 (8.7%)	8 (27.6%)	0.155	0.209 (0.047-1.783)	0.181

a Adjusted for age at operation and sex unless otherwise specified; characteristics of sporadic MPTCs were used as references.

b Adjusted for age at operation.

c Adjusted for sex.

d Not available.

The numbers in bold indicate statistical significance.

In young adults aged 19-30 years at diagnosis (**Table 3**), statistically significant differences in the radiogenic subgroup were found for older age (b = 19.241, p = 5.27E-07), longer follow-up period (b = 1.501, p = 2.00E-06) and lower frequency of concomitant chronic thyroiditis (OR = 0.421, p = 0.030). Patients with radiogenic MPTC were more likely to receive RIT (OR = 2.391, p = 0.039) and more RIT cycles (OR = 3.583, p = 6.61E-04). As in the whole group, RIT response was more favorable in the radiogenic subgroup (OR = 6.797, p = 0.024), and the frequency of excellent response was higher (OR = 6.914, p = 0.025). Since all recurrences occurred only in this age subgroup, all observations from the whole-group analysis were reproduced: RAI-R metastases were absent among 3 recurrences in the radiogenic group, and 2/2 occurred in the sporadic group, with statistical estimates identical to those presented in **Table 1**. The higher frequency of encapsulated tumors (OR = 2.487, p = 0.082), and less frequent solid-trabecular growth pattern (OR = 0.459, p = 0.065), oncocytic changes (OR = 0.490, p = 0.055), N1b tumors (OR = 0.292, p = 0.080) and concomitant Graves disease (OR = 0.065, p = 0.060) for the radiogenic MPTCs were suggestive.

**Table 3:** Characteristics of the radiogenic and sporadic papillary thyroid microcarcinomas in young adult patients aged 19-30 years.

Parameters	RAD, n = 113	SPOR, n = 54	p-value	OR, b or HR (95% CI)	p-value
	number or value (% or IQR)	number or value (% or IQR)	univariate	multivariate^a^	
**Sex** (F/M; %M; F:M ratio, ref=F)	90/23; 20.4%; 3.9:1	42/12; 22.2%; 3.5:1	0.840	1.397 (0.580-3.364)^b^	0.456
**Age at operation**, years	27.7 (25.1-29.2)	23.5 (21.7-26.4)	**2.17E-06**	**19.241 (11.967-26.514)** ^c^	**5.27E-07**
**Tumor size**, mm	7 (6-9)	7 (6-9)	0.340	0.917 (-0.399-2.233)	0.171
1 - 5 mm	19 (16.8%)	12 (22.2%)	0.403	0.592 (0.243-1.443)	0.249
6 - 10 mm	94 (83.2%)	42 (77.8%)	0.403	1.688 (0.693-4.114)	0.249
**Complete tumor capsule**	21 (18.6%)	7 (13.0%)	0.507	2.487 (0.889-6.957)	0.082
**Dominant growth pattern**			0.517	0.661 (0.338-1.291)	0.225
papillary	63 (55.8%)	27 (50.0%)	0.511	1.313 (0.649-2.656)	0.449
follicular	26 (23.0%)	11 (20.4%)	0.843	1.529 (0.638-3.668)	0.341
solid-trabecular	24 (21.2%)	16 (29.6%)	0.250	0.459 (0.201-1.051)	0.065
**Ki-67 labeling index**	n = 20; 6.3 (4.3-8.3)	n = 18; 5.0 (3.1-6.8)	0.111	0.359 (-0.368-1.086)	0.322
0 - 5%	7 (35.0%)	10 (55.6%)	0.328	0.717 (0.108-4.774)	0.731
>5 - 10%	9 (45.0%)	7 (38.9%)	0.752	1.141 (0.179-7.251)	0.889
>10%	4 (20.0%)	1 (5.6%)	0.344	1.747 (0.132-23.153)	0.672
**BRAF^V600E^-positive**	n = 21; 12 (57.1%)	n = 20; 12 (60.0%)	1.000	0.414 (0.080-2.128)	0.291
**Oncocytic changes**	44 (38.9%)	27 (50.0%)	0.185	0.490 (0.236-1.015)	0.055
**Multifocality**	26 (23.0%)	17 (31.5%)	0.260	0.698 (0.319-1.527)	0.368
**Lymphatic/vascular invasion**	31 (27.4%)	18 (33.3%)	0.470	0.751 (0.349-1.616)	0.464
**Extrathyroidal extension (any)**	15 (13.3%)	8 (14.8%)	0.813	1.171 (0.424-3.230)	0.761
**N category (N1)**	22 (19.5%)	19 (35.2%)	**0.035**	0.552 (0.249-1.227)	0.145
N1a	18 (15.9%)	12 (22.2%)	0.389	0.821 (0.336-2.003)	0.664
N1b	4 (3.5%)	7 (13.0%)	**0.040**	0.292 (0.074-1.157)	0.080
**M category (M1)**	2 (1.8%)	1 (1.9%)	1.000	0.779 (0.102-5.935)	0.809
**pT**					
pT1a	112 (99.1%)	53 (98.1%)	0.543	1.249 (0.065-23.831)	0.883
pT3b	1 (0.9%)	1 (1.9%)	0.543	0.799 (0.091-7.041)	0.840
**Invasiveness score**	1 (0-1)	1 (0-2)	0.069	0.690 (0.363-1.313)	0.259
0	51 (45.1%)	21 (38.9%)	0.506	1.287 (0.628-2.638)	0.491
1	36 (31.9%)	14 (25.9%)	0.475	1.166 (0.533-2.549)	0.700
2	19 (16.8%)	11 (20.4%)	0.667	0.709 (0.287-1.752)	0.457
3	6 (5.3%)	5 (9.3%)	0.337	0.801 (0.196-3.272)	0.757
4	1 (0.9%)	3 (5.6%)	0.100	0.349 (0.051-2.363)	0.281
**Thyroid surgery volume**					
total thyroidectomy	99 (87.6%)	44 (81.5%)	0.347	2.045 (0.775-5.401)	0.149
organ-preserving operation	14 (12.4%)	10 (18.5%)	0.347	0.489 (0.185-1.291)	0.149
**LN dissection performed**	53 (46.9%)	27 (50.0%)	0.742	0.991 (0.490-2.004)	0.979
level ≥ 6	36 (31.9%)	19 (35.2%)	0.726	0.874 (0.415-1.842)	0.724
level 1 – 5	17 (15.0%)	8 (14.8%)	1.000	1.235 (0.456-3.346)	0.678
**RIT performed**	91 (80.5%)	37 (68.5%)	0.117	**2.391 (1.046-5.468)**	**0.039**
**RIT cycles**	1 (1-1)	1 (0-1)	**0.015**	**3.583 (1.719-7.470)**	**6.61E-04**
**Cumulative RI activity,** MBq	3850 (2533-4360)	4360 (3888-4390)	**0.020**	-0.147 (-0.532-0.239)	0.454
**RIT response**	n = 117	n = 37	**0.005**	**6.797 (1.284-35.984)**	**0.024**
RAI-R recurrence *vs* other	0	2 (5.4%)	0.082	0.122 (0.008-1.787)	0.125
excellent *vs* other	88 (96.7%)	31 (83.8%)	**0.017**	**6.914 (1.274-37.522)**	**0.025**
**Follow-up**, years	6.4 (3.0-11.5)	3.0 (1.5-5.3)	**2.06E-05**	**1.501 (0.896-2.106)**	**2.00E-06**
**LN recurrence after 6 months**	3 (2.1%)	2 (3.7%)	0.628^d^	0.760 (0.133-4.921)^e^	0.773
**Time to recurrence**, yrs	1.2 (1.2-1.2)	1.1 (0.9-1.3)	1.000	0.222 (-3.603-4.047)	0.596
**Recurrent metastases:**	n = 3	n = 2			
Dominant growth pattern			1.000	NA^f^	0.668
papillary	2 (66.7%)	1 (50.0%)	1.000	1.155 (0.002-567.739)	0.964
follicular	1 (33.3%)	0	1.000	16.740 (0.011-inf^g^)	0.450
solid-trabecular	0	1 (50.0%)	0.400	0.240 (0.000-117.849)	0.652
Ki67 labeling index	n = 1; 0.8	n = 2; 2.8 (2.5-3.1)	NA	NA	NA
Oncocytic changes	1 (33.3%)	2 (100%)	0.400	0.031 (0.000-36.002)	0.335
Cystic changes	2 (66.7%)	1 (50.0%)	1.000	72.239 (0.006-inf)	0.376
**Additional thyroid cancer**	0	1 (1.9%)	0.323	0.079 (0.005-1.351)	0.080
**Additional nodular disease**	21 (18.6%)	8 (14.8%)	0.664	1.232 (0.471-3.223)	0.670
**Additional Graves disease**	0	2 (3.7%)	0.103	0.065 (0.004-1.127)	0.060
**Chronic thyroiditis**	28 (24.8%)	21 (38.9%)	0.071	**0.421 (0.193-0.919)**	**0.030**

a Adjusted for age at operation and sex unless otherwise specified; characteristics of sporadic MPTCs were used as references.

b Adjusted for age at operation.

c Adjusted for sex.

d The log-rank test.

e The Firth’s penalized proportional hazard model.

f Not available.

g Infinity.

The numbers in bold indicate statistical significance.

**Figure 2:**
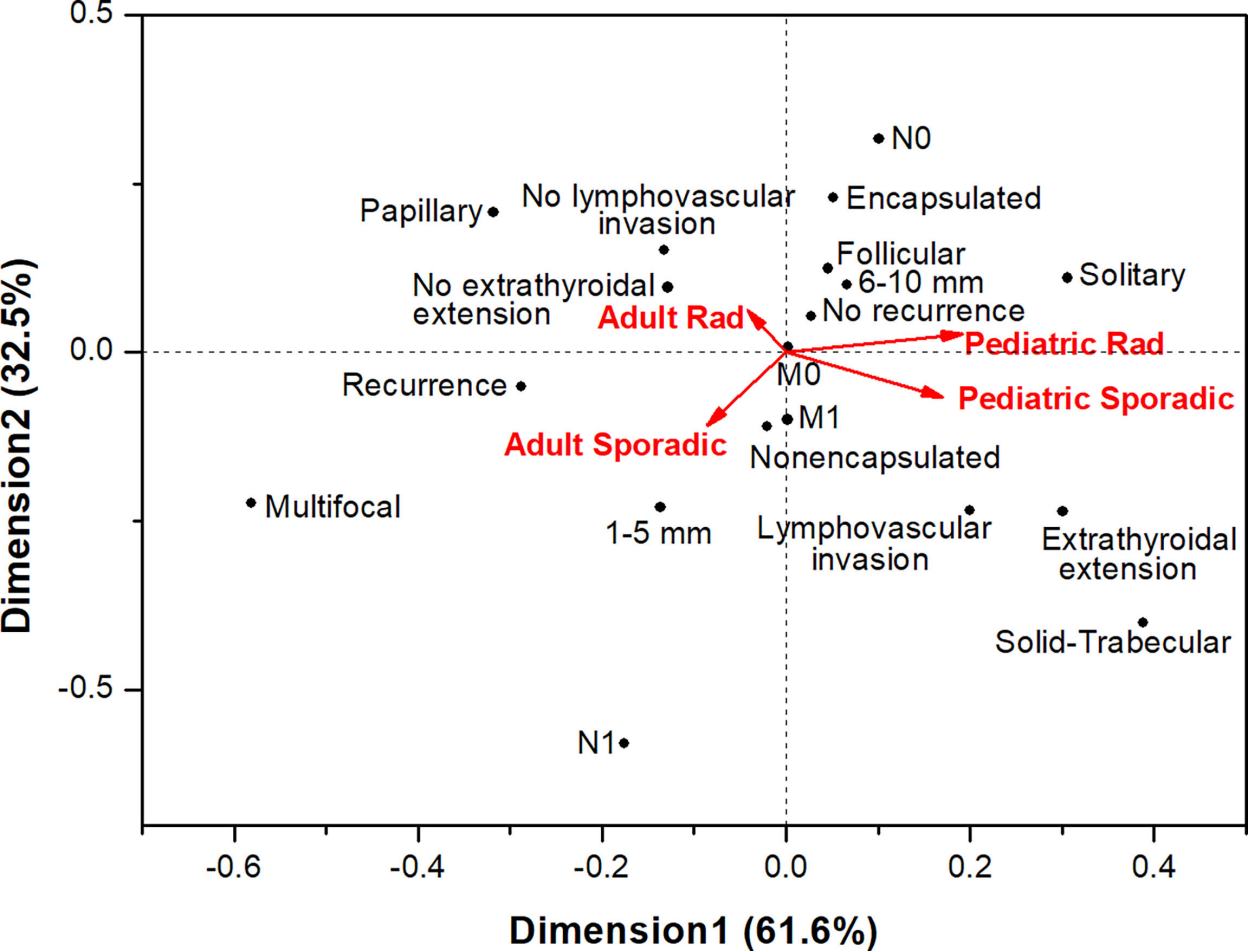
Correspondence analysis of the associations of papillary thyroid microcarcinomas of different etiology from pediatric ( ≤ 18-year-old.) and young adult (19-30-year-old) patients with the major histopathological characteristics, tumor invasive features and recurrence.

We also performed correspondence analysis to visualize the similarities or differences in structural, invasive and prognostic characteristics of MPTCs of the two etiological forms in patients of different age at diagnosis (**Figure 2**). The results clearly demonstrated a pronounced similarity between pediatric radiogenic and sporadic MPTCs (note the acute angle between these groups). MPTCs from young adults showed differences from pediatric tumors (the obtuse angles between any young adult *vs*. any pediatric subgroup). The radiogenic and sporadic MPTCs from young adult patients also looked different (note the right angle between these subgroups). In agreement with the multivariate analysis (see **Tables 2**, **3**), no evidence in support of more aggressive tumor behavior or poorer prognosis were observed for the radiogenic MPTCs of any age subgroup: their localizations on the graph were more likely to be in the opposite directions to the invasive or prognostically less favorable parameters, in contrast to sporadic MPTCs.

### MPTCs size subgroups

The radiogenic and sporadic tumors sized up to 5 mm (**Table 4**) displayed practically no differences between the baseline, histopathological and clinical characteristics except for the less frequent central lymph node dissection (OR = 0.163, p = 0.031) in, and longer follow-up period (OR = 1.935, p = 0.001) of patients with radiogenic MPTC.

**Table 4:** Characteristics of the radiogenic and sporadic papillary thyroid microcarcinomas sized up to 5 mm.

Parameters	RAD, n = 22	SPOR, n = 17	p-value	OR, b or HR (95% CI)	p-value
	number or value (% or IQR)	number or value (% or IQR)	univariate	multivariate^a^	
**Sex** (F/M; %M; F:M ratio, ref=F)	18/3; 18.2%; 6.0:1	13/4; 23.5%; 3.3:1	0.709	1.147 (0.205-6.411)^b^	0.876
**Age at operation**, years	27.3 (24.6-28.8)	22.5 (18.3-27.7)	0.095	19.151 (-2.865-41.166)^c^	0.086
**Tumor size**, mm, median	5 (5-5)	5 (5-5)	0.258	0.021 (-0.673-0.715)	0.952
1 - 5 mm	22 (100%)	17 (100%)	NA^d^	NA	NA
6 - 10 mm	0	0	NA	NA	NA
**Complete tumor capsule**	2 (9.1%)	1 (5.9%)	1.000	3.572 (0.211-60.486)	0.378
**Dominant growth pattern**			1.000	0.861 (0.243-3.043)	0.816
papillary	8 (36.4%)	7 (41.2%)	1.000	1.269 (0.294-5.477)	0.750
follicular	6 (27.3%)	4 (23.5%)	1.000	0.980 (0.219-4.382)	0.979
solid-trabecular	8 (36.4%)	6 (35.3%)	1.000	0.807 (0.196-3.316)	0.766
**Ki-67 labeling index**, median	n = 5; 6.0 (4.2-6.7)	n = 4; 2.9 (2.5-5.2)	0.462	0.279 (-1.155-1.713)	0.638
0 - 5%	2 (40.0%)	3 (75.0%)	0.524	0.312 (0.011-8.716)	0.493
>5 - 10%	3 (60.0%)	1 (25.0%)	0.524	3.208 (0.115-89.714)	0.493
>10%	0	0	NA	NA	NA
**BRAF^V600E^-positive**	n = 5; 4 (80.0%)	n = 4; 3 (75.0%)	1.000	0.679 (0.023-19.639)	0.821
**Oncocytic changes**	7 (31.8%)	8 (47.1%)	0.508	0.378 (0.089-1.604)	0.187
**Multifocality**	7 (31.8%)	2 (11.8%)	0.251	2.975 (0.552-16.036)	0.205
**Lymphatic/vascular invasion**	5 (22.7%)	3 (17.6%)	1.000	1.914 (0.324-11.306)	0.474
**Extrathyroidal extension (any)**	2 (9.1%)	2 (11.8%)	1.000	1.038 (0.146-7.372)	0.970
**N category (N1)**	6 (27.3%)	5 (29.4%)	1.000	1.058 (0.239-4.691)	0.941
N1a	3 (13.6%)	3 (17.6%)	1.000	0.645 (0.114-3.664)	0.621
N1b	3 (13.6%)	2 (11.8%)	1.000	1.522 (0.229-10.107)	0.664
**M category (M1)**	1 (4.5%)	0	1.000	2.941 (0.131-65.945)	0.497
**pT**					
pT1a	22 (100%)	16 (94.1%)	0.436	3.257 (0.178-59.560)	0.426
pT3b	0	1 (5.9%)	0.436	0.307 (0.017-5.615)	0.426
**Invasiveness score**	1 (0-2)	0 (0-1)	0.507	2.114 (0.583-7.673)	0.255
0	9 (40.9%)	9 (52.9%)	0.528	0.503 (0.125-2.020)	0.333
1	7 (31.8%)	5 (29.4%)	1.000	1.083 (0.256-4.583)	0.914
2	4 (18.2%)	2 (11.8%)	0.679	1.805 (0.283-11.496)	0.532
3	2 (9.1%)	1 (5.9%)	1.000	1.822 (0.204-16.280)	0.591
**Thyroid surgery volume**					
total thyroidectomy	17 (77.3%)	16 (94.1%)	0.206	0.281 (0.027-2.872)	0.284
organ-preserving operation	5 (22.7%)	1 (5.9%)	0.206	2.466 (0.358-16.984)	0.359
**LN dissection performed**	8 (36.4%)	9 (52.9%)	0.345	0.307 (0.080-1.181)	0.086
level ≥ 6	5 (22.7%)	7 (41.2%)	0.299	**0.163 (0.031-0.847)**	**0.031**
level 1 – 5	3 (13.6%)	2 (11.8%)	1.000	1.283 (0.164-10.031)	0.812
**RIT performed**	17 (77.3%)	11 (64.7%)	0.482	1.996 (0.454-8.781)	0.360
**RIT cycles**	1 (1-1)	1 (0-1)	0.109	3.198 (0.791-12.928)	0.103
**Cumulative RI activity,** MBq	3924 (3509-4436)	4360 (3926-4360)	0.924	0.370 (-0.374-1.114)	0.315
**RIT response**	n = 17	n = 11	1.000	0.498 (0.042-5.910)	0.581
RAI-R recurrence *vs* other	0	0	NA	NA	NA
excellent *vs* other	14 (82.4%)	10 (90.9%)	1.000	0.488 (0.041-5.886)	0.488
**Follow-up**, years	6.4 (2.7-9.1)	2.7 (0.7-4.4)	**0.019**	**1.935 (0.827-3.043)**	**0.001**
**LN recurrence after 6 months**	0	0	NA	NA	NA
**Additional thyroid cancer**	0	0	NA	NA	NA
**Additional nodular disease**	2 (9.1%)	6 (35.3%)	0.059	0.212 (0.038-1.178)	0.076
**Additional Graves disease**	0	2 (11.8%)	0.184	0.144 (0.008-2.556)	0.187
**Chronic thyroiditis**	5 (22.7%)	4 (23.5%)	1.000	0.717 (0.143-3.585)	0.685

a Adjusted for age at operation and sex unless otherwise specified; characteristics of sporadic MPTCs were used as references.

b Adjusted for age at operation.

c Adjusted for sex.

d Not available.

The numbers in bold indicate statistical significance.

**Figure 3:**
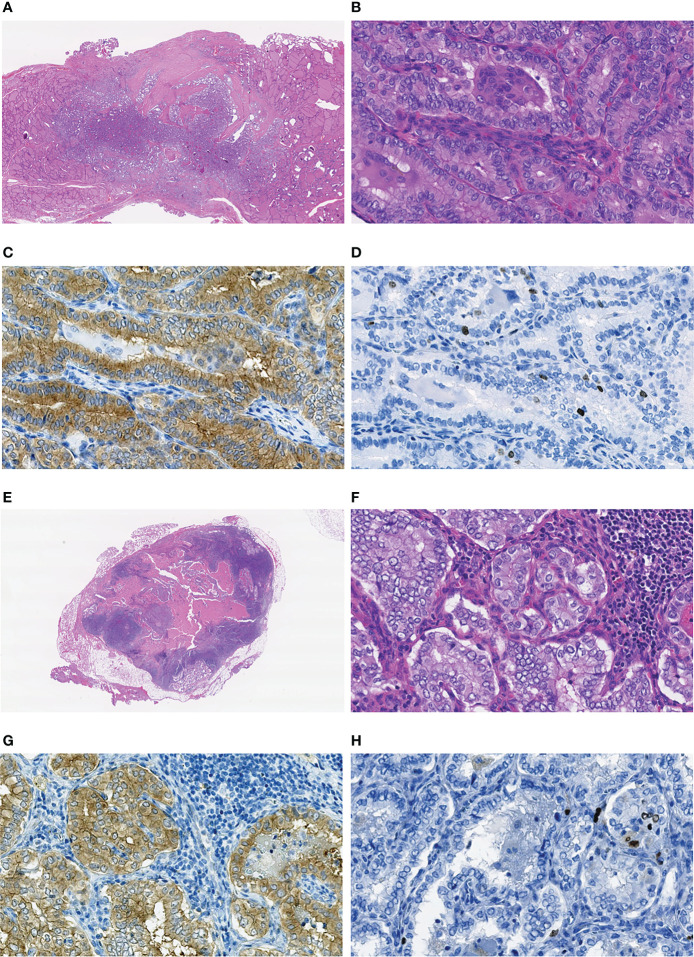
Sporadic recurrent MPTC sized 8 mm (pT1aN0M0 at the first operation) removed from a 22-year-old male patient: **(A–D)** primary tumor, **(E–H)** the RAI-R recurrent metastasis. **(A)** Nonencapsulated primary tumor with follicular-papillary (conventional) growth pattern with extrathyroidal extension to the connective tissue, H&E, 15X magnification. **(B)** Fragment of the primary tumor with papillary structures featuring tall cell areas and oncocytic changes, H&E, 400X magnification. **(C)** Primary tumor: positive IHC reaction with the anti-BRAF (mutated V600E) antibody, 400X magnification. **(D)** Primary tumor: IHC reaction with Ki67 (Clone MIB-1) antibody (Ki67 LI 3.1%), 400X magnification. **(E)** RAI-R recurrent metastasis with cystic changes removed 1.3 years after the first surgery, H&E, 15X magnification. **(F)** Fragment of the RAI-R recurrent metastasis with solid structure and oncocytic changes, H&E, 400X magnification. **(G)** fragment of the RAI-R recurrent metastasis: positive IHC reaction with anti-BRAF (mutated V600E) antibody, 400X magnification. **(H)** RAI-R recurrent metastasis: IHC reaction with Ki67 (Clone MIB-1) antibody (Ki67 LI 3.3%), 400X magnification.

In MPTCs of larger size (6-10 mm), statistically significant differences between the radiogenic and sporadic series were found for a number of parameters (**Table 5**). Those included the older age of patients with radiogenic MPTCs (b = 28.445, p = 2.62E-07), less frequent oncocytic changes (OR = 0.390, p = 0.010), multifocality (OR = 0.365, p = 0.017), nodal disease (OR = 0.419, p = 0.028; for N1b, OR = 0.229, p = 0.025), lower invasiveness score (OR = 0.474, p = 0.015; and more frequent invasiveness score 0, OR = 2.095, p = 0.035), and less frequent chronic thyroiditis (OR = 0.373, p = 0.011). The radiogenic MPTCs were also associated with lower cumulative RI activity (b = -0.384, p = 0.021), yet better excellent RIT response (OR = 94.032, p = 0.003), suggestively less frequent RAI-R (OR = 0.066, p = 0.073), and longer follow-up (b = 1.717, p = 8.23E-09). Again, all two RAI-R recurrences measuring 6 and 8 mm were among the sporadic MPTCs. In **Figure 3** we present one of these tumors harboring the *BRAFV^600E^* mutation and featuring oncocytic changes in both primary tumor and recurrent metastasis epithelial cells.

**Table 5:** Characteristics of the radiogenic and sporadic papillary thyroid microcarcinomas sized 6-10 mm.

Parameters	RAD, n = 114	SPOR, n = 66	p-value	OR, b or HR (95%CI)	p-value
	number or value (% or IQR)	number or value (% or IQR)	univariate	multivariate^a^	
**Sex** (F/M; %M; F:M ratio, ref=F)	84/30, 26.3%, 2.8:1	50/16, 24.2%, 3.1:1	0.860	1.537 (0.719-3.285)^b^	0.267
**Age at operation**, years	26.0 (22.0-28.7)	21.3 (17.0-23.9)	**3.59E-07**	**28.445 (17.964-38.926)** ^c^	**2.62E-07**
**Tumor size**, mm	8 (7-9)	8 (7-10)	0.528	-0.306 (-1.317-0.705)	0.551
1 - 5 mm	0	0	NA^d^	NA	NA
6 - 10 mm	141 (100%)	66 (100%)	NA	NA	NA
**Complete tumor capsule**	23 (20.2%)	11 (16.7%)	0.693	1.399 (0.600-3.266)	0.437
**Dominant growth pattern**			0.262	0.920 (0.494-1.713)	0.794
papillary	63 (55.3%)	31 (47.0%)	0.353	0.963 (0.493-1.880)	0.911
follicular	26 (22.8%)	13 (19.7%)	0.709	1.423 (0.633-3.196)	0.393
solid-trabecular	25 (21.9%)	22 (33.3%)	0.113	0.764 (0.362-1.613)	0.480
**Ki-67 labeling index**, median	n = 38; 5.5 (2.9-790)	n = 32; 5.0 (3.7-8.2)	0.861	-0.126 (-0.697-0.445)	0.660
0 - 5%	13 (48.1%)	17 (53.1%)	0.796	0.819 (0.259-2.592)	0.735
>5 - 10%	10 (37.0%)	9 (28.1%)	0.579	1.387 (0.408-4.715)	0.600
>10%	4 (14.8%)	6 (18.8%)	0.741	0.881 (0.218-3.559)	0.859
**BRAFV600E-positive**	n = 29; 10 (34.5%)	n = 34; 17 (50.0%)	0.307	0.332 (0.100-1.102)	0.072
**Oncocytic changes**	39 (34.2%)	31 (47.0%)	0.113	**0.390 (0.191-0.797)**	**0.010**
**Multifocality**	20 (17.5%)	18 (27.3%)	0.133	**0.365 (0.159-0.835)**	**0.017**
**Lymphatic/vascular invasion**	35 (30.7%)	26 (39.4%)	0.256	0.713 (0.360-1.413)	0.333
**Extrathyroidal extension (any)**	17 (14.9%)	14 (21.2%)	0.309	0.814 (0.352-1.883)	0.630
**N category (N1)**	20 (17.5%)	21 (31.8%)	**0.042**	**0.419 (0.194-0.908)**	**0.028**
N1a	17 (14.9%)	12 (18.2%)	0.674	0.674 (0.280-1.625)	0.380
N1b	3 (2.6%)	9 (13.6%)	**0.010**	**0.229 (0.063-0.829)**	**0.025**
**M category (M1)**	1 (0.9%)	2 (3.0%)	0.555	0.331 (0.046-2.384)	0.272
**pT**					
pT1a	113 (99.1%)	66 (100%)	1.000	0.608 (0.036-10.267)	0.730
pT3b	1 (0.9%)	0	1.000	1.645 (0.097-27.799)	0.730
**Invasiveness score**	1 (0-2)	1 (0-2)	**0.012**	**0.474 (0.259-0.867)**	**0.015**
0	54 (47.4%)	22 (33.3%)	0.085	**2.095 (1.053-4.165)**	**0.035**
1	34 (29.8%)	21 (31.8%)	0.867	0.792 (0.390-1.609)	0.519
2	20 (17.5%)	13 (19.7%)	0.842	0.823 (0.354-1.913)	0.651
3	5 (4.4%)	6 (9.1%)	0.215	0.414 (0.110-1.563)	0.193
4	1 (0.9%)	4 (6.1%)	0.061	0.238 (0.036-1.467)	0.123
**Thyroid surgery volume**					
total thyroidectomy	101 (88.6%)	55 (83.3%)	0.365	1.744 (0.677-4.491)	0.249
organ-preserving operation	13 (11.4%)	11 (16.7%)	0.365	0.574 (0.223-1.477)	0.249
**Lymph node dissection performed**	51 (44.7%)	30 (45.5%)	1.000	0.843 (0.473-1.628)	0.611
level ≥ 6	33 (28.9%)	19 (28.8%)	1.000	0.751 (0.360-1.567)	0.446
level 1 – 5	18 (15.8%)	11 (16.7%)	1.000	1.078 (0.449-2.586)	0.867
**RIT performed**	87 (76.3%)	48 (72.2%)	0.597	1.012 (0.480-2.135)	0.975
**RIT cycles**	1 (1-1)	1 (0-1)	0.688	1.189 (0.628-2.253)	0.595
**Cumulative RI activity,** MBq	3768 (2402-4340)	4204 (3405-4436)	**0.011**	**-0.384 (-0.710- -0.058)**	**0.021**
**RIT response**	n = 87	n = 48	**0.002**	NA	NA
RAI-R recurrence *vs* other	0	2 (4.2%)	0.125	0.066 (0.003-1.293)	0.073
excellent *vs* other	87 (100%)	42 (87.5%)	**0.002**	**94.032 (4.462-inf** ^e^ **)**	**0.003**
**Follow-up**, years	9.0 (3.5-15.2)	4.3 (2.1-8.4)	**6.54E-05**	**1.717 (1.157-2.276)**	**8.23E-09**
**LN recurrence after 6 months**	3 (2.1%)	2 (3.0%)	0.829^f^	0.608 (0.106-3.931)^g^	0.601
**Time to recurrence**, yrs	1.2 (1.2-1.2)	1.1 (0.9-1.3)	1.000	0.109 (-3.290-3.508)	0.753
**Recurrent metastases**	n = 3	n = 2	0.655	0.506 (0.072-3.573)	0.494
Dominant growth pattern			1.000	NA	NA
papillary	2 (66.7%)	1 (50.0%)	1.000	1.155 (0.002-567.739)	0.964
follicular	1 (33.3%)	0	1.000	16.740 (0.011-inf)	0.450
solid-trabecular	0	1 (50.0%)	0.400	0.240 (0.000-117.849)	0.652
Ki67 labeling index	n = 1; 0.8	n = 2; 2.8 (2.5-3.1)	0.221	NA	NA
Oncocytic changes	1 (33.3%)	2 (100%)	0.400	0.031 (0.000-36.002)	0.335
Cystic changes	2 (66.7%)	1 (50.0%)	1.000	72.239 (0.006-inf)	0.376
**Additional thyroid cancer**	0	1 (1.5%)`	0.367	0.064 (0.003-1.187)	0.065
**Additional nodular disease**	22 (19.3%)	7 (10.6%)	0.145	2.029 (0.763-5.392)	0.156
**Additional Graves disease**	0	1 (1.5%)	0.367	0.191 (0.012-3.150)	0.247
**Chronic thyroiditis**	25 (21.9%)	25 (37.9%)	**0.025**	**0.373 (0.174-0.798)**	**0.011**

a Adjusted for age at operation and sex unless otherwise specified; characteristics of sporadic PTCs were used as references.

b Adjusted for age at operation.

c Adjusted for sex.

d Not available.

e Infinity.

f The log-rank test.

g The Firth’s penalized proportional hazard model.

The numbers in bold indicate statistical significance.

**Figure 4:**
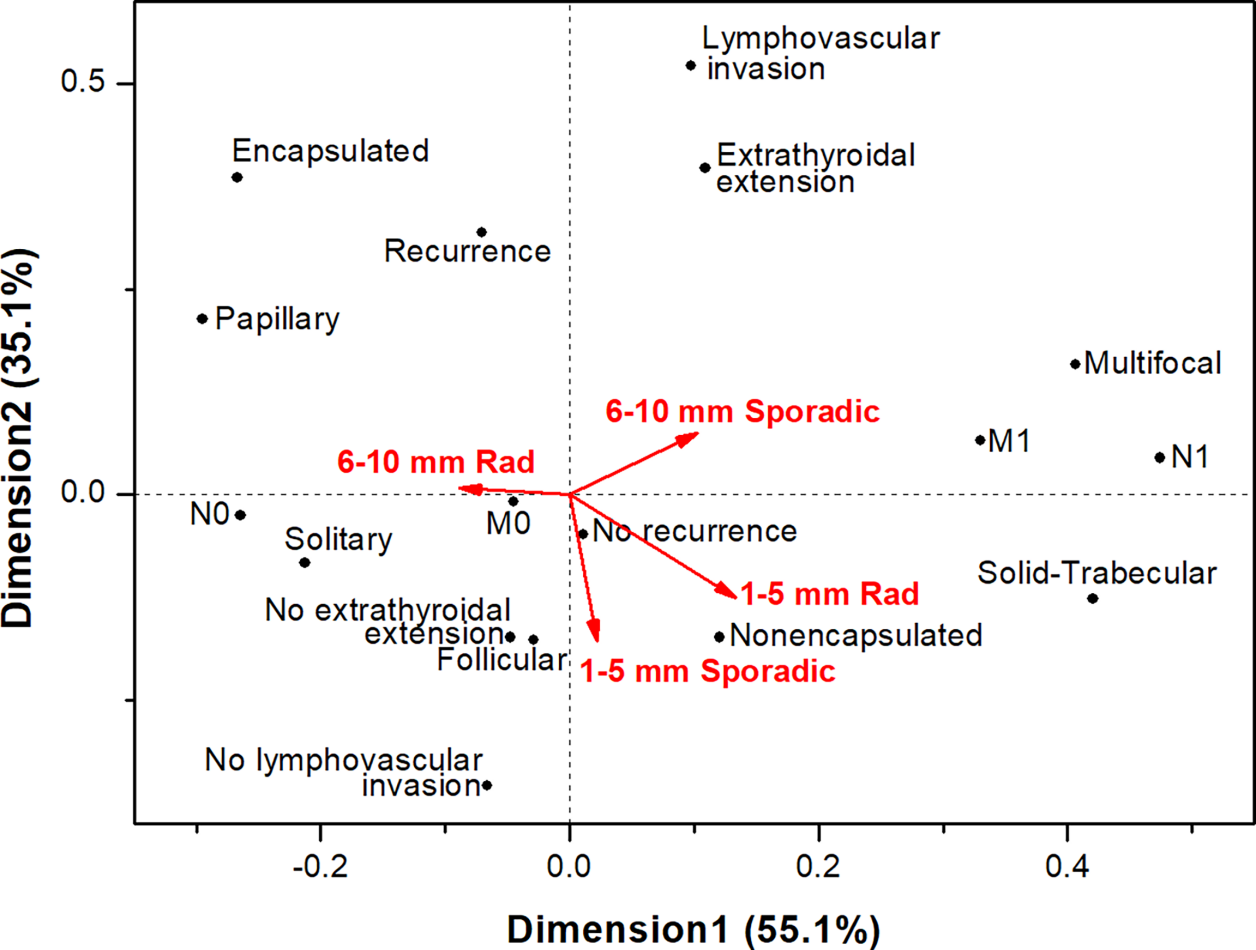
Correspondence analysis of the associations of papillary thyroid microcarcinomas of different etiology sized 1-5 mm and 6-10 mm with the major histopathological characteristics, tumor invasive features and recurrence.

Correspondence analysis of MPTCs of different etiology and size subgroups (**Figure 4**) confirmed that these tumors did not display aggressive phenotype overall (note the upper right quadrant, where the aggressive characteristics are located). The radiogenic MPTCs measuring 6-10 mm appeared different from other subgroups (note the obtuse angles between this and other subgroups), yet without signs of association with aggressive features. The subgroups of radiogenic and sporadic MPTCs measuring 1-5 mm were rather similar in their properties (note the acute angle between those) without evidence of tumor aggressiveness or poorer prognosis. The sporadic MPTCs sized 6-10 mm, but not the radiogenic ones, weakly tended to associate with more aggressive tumor phenotype.

### Parameters potentially distinguishing the radiogenic and sporadic MPTCs

To further delineate the characteristics (parameters) that might be associated with the radiogenic or sporadic etiology of MPTCs in the current series, we applied several statistical algorithms of variable selection (**Table 6**). The older age at surgery, the less frequent nodal disease, oncocytic changes, multifocality, and male sex were the most likely characteristics that were associated with radiogenic MPTC. Other potentially relevant variables might be excellent RIT response and less frequent lymphovascular invasion. Hence, for any algorithm, no evidence of association of the radiogenic MPTCs with aggressive tumor behavior or poorer prognosis, as compared to sporadic MPTCs, was found.

**Table 6:** The variables potentially contributing to the differences between the non-incidental radiogenic and sporadic papillary thyroid microcarcinomas selected by different statistical methods.

Parameters	OR (95% CI)	p-value	AIC^a^	AUC^b^ training	AUC validation
*Method 1: adaptive lasso*			144.4	0.948	0.894
**Age at operation**, years	1.343 (1.260-1.434)	< 0.001			
**Excellent RIT response**	3.839 (0.919-16.032)	0.065			
**N category (N1)**	0.586 (0.213-1.615)	0.302			
**Oncocytic changes**	0.674 (0.256-1.778)	0.426			
**Multifocality**	0.671 (0.238-1.889)	0.450			
**Sex (M)**	1.356 (0.474-3.883)	0.570			
*Method 2: best subset*			146.5	0.943	0.908
**Age at operation**, years	1.359 (1.265-1.460)	< 0.001			
**Oncocytic changes**	0.430 (0.159-1.165)	0.097			
**Multifocality**	0.606 (0.220-1.673)	0.334			
**Sex (M)**	1.624 (0.498-5.302)	0.422			
**N category (N1)**	0.718 (0.249-2.071)	0.540			
**Tumor size 6-10 mm**	1.214 (0.326-4.519)	0.773			
**Lymphatic/vascular invasion**	0.896 (0.319-2.517)	0.843			
*Method 3: pruned forward selection*			147.6	0.942	0.913
**Age at operation**, years	1.365 (1.253-1.488)	< 0.001			
**Oncocytic changes**	0.390 (0.143-1.060)	0.065			
**N category (N1)**	0.456 (0.160-1.296)	0.141			
**Excellent RIT response**	2.494 (0.640-9.716)	0.188			
**Sex (M)**	2.025 (0.660-6.219)	0.218			
*Method 4: adaptive elastic net*			155.8	0.936	0.918
**Age at operation**, years	1.345 (1.251-1.447)	< 0.001			
**Oncocytic changes**	0.275 (0.106-0.710)	0.008			
**N category (N1)**	0.713 (0.273-1.862)	0.490			
**Multifocality**	0.740 (0.294-1.863)	0.522			
**Sex (M)**	1.293 (0.457-3.661)	0.557			
**Lymphatic/vascular invasion**	0.798 (0.305-2.092)	0.646			

a Akaike information criterion.

b Area under curve.

## Discussion

A link between previous radiation exposure and MPTC has been established in atomic bomb victims, in whom an increased dose-dependent risk for developing tumors has been observed (38). However, despite the 2015 ATA Guidelines mention of radiation history as a potential contraindication to less extensive thyroid surgery for patients with low-risk PTC (22), there is little evidence from clinical and histopathological studies that would justify such warning. In part, this may be due to the relative infrequency of patients with thyroid cancer who had been irradiated at a young age. We therefore considered it important to perform a study of MPTCs from patients of comparable age with proven previous internal radiation exposure at the age below 18 or without such. The radiogenic MPTCs in our work were from patients from the high-risk group of subjects who lived in the northern, most radiocontaminated by the Chornobyl fallouts, regions of Ukraine. The individual radiation doses to the thyroid were calculated and confirmed for all of them.

The rationale for focusing the study on young patients (here, aged up to 30 years) was that our previous investigation found higher aggressiveness of radiogenic PTCs, especially in children and adolescents (25, 26). However, the mentioned studies did not specifically address the effect of tumor size, and therefore whether these findings are applicable to MPTCs remained unclear. It is also important that more than 35 years after the Chornobyl accident, a sufficient control group of patients with MPTCs who lived in the same regions but had not been exposed to radiation became available.

Our analysis of MPTCs from two etiological groups detected several statistically significant differences including older age of patients in the radiogenic series, less frequent oncocytic changes, lymph node metastases and concomitant chronic thyroiditis (see **Table 1**). RIT results were better in patients with radiogenic MPTCs, excellent response was more frequent in this group, and no RAI-R metastases were observed. No signs of higher aggressiveness or poorer prognosis in the radiogenic MPTCs were found.

Comparison of MPTCs in the pediatric subgroup did not reveal significant differences in the clinical and histopathological characteristics between the radiogenic and sporadic pediatric MPTCs (see **Table 2** and **Figure 2**), except for the lower frequency of oncocytic changes in the radiogenic MPTCs, which is in line with data obtained earlier for PTCs of any size (34). It is noteworthy that with median sizes of 7 and 8 mm of the radiogenic and sporadic MPTCs, respectively, and the presence of lymph node metastases in about 20% of patients (see **Table 2**), which is associated with worse prognosis according to the literature (39–41), no one pediatric patient in both groups developed recurrence during the 19.4 and 6.3 years, respectively, of median follow-up.

In young adult patients aged 19-30 years (see **Table 3**), no evidence in support of higher aggressiveness of the radiogenic MPTCs was found. Regional recurrences occurred in isolated cases (2.1% radiogenic and 3.7% sporadic), but in the sporadic subgroup, in contrast to the radiogenic subgroup, these were BRAF^V600E^-positive and RAI-R (shown in **Figure 3**). As in the whole group, young adult patients with radiogenic MPTCs were more likely to achieve complete remission (excellent response) after RIT (see **Table 3**).

In the analysis by tumor size, a number of differences between the radiogenic and sporadic MPTCs were observed for the 6-10 mm subgroups (see **Tables 4**, **5**, **Figure 4**). Again, these differences were rather suggestive of a less aggressive phenotype of the radiogenic MPTC as compared to sporadic. Virtually no difference was seen between the radiogenic and sporadic MPTCs measuring 1-5 mm.

Thus, in young patients in the whole group or age or size subgroups, no clinical and histopathological parameters pointing at more aggressive behavior of radiogenic MPTCs were found. With regard to clinical implications, it would be pertinent to suggest that the extent of surgery for MPTC in internally irradiated patients should not be principally different from that in non-exposed patients aged up to 30 years; in other words, etiological considerations may be omitted. Furthermore, patients with radiogenic MPTCs were more likely to achieve complete remission and did not develop RAI-R recurrent metastases.

Notwithstanding the overall indolent clinical presentation of MPTCs, in both etiological groups there were tumors with signs of morphological aggressiveness: multifocal growth, lymphatic/vascular invasion, extrathyroidal extension (almost always minimal), lymph node metastases and even distant metastases (see **Table 1**). These morphological characteristics have been identified as negative prognostic factors for MPTCs in many studies (42–47), and, in turn, have been correlated with a larger MPTC size (48–51). Recurrences were observed in isolated cases of our study too, and occurred in the subgroups of patients of older age (here, the young adults) with MPTCs measuring 6-10 mm. Hence, regardless of MPTC etiology, our study additionally confirmed that the “small tumors” should be stratified into the low-, intermediate-, and high-risk ones (40, 47, 52) both in pediatric and adult patients in line with existing guidelines (22, 53).

It is worth noting that to draw more attention to the problem of treatment of microcarcinoma, a somewhat obscure and not necessarily correct – in our opinion – term “overdiagnosis” has been introduced and extensively used (3–5, 10, 11). Indeed, the improvement of ultrasound diagnostic methods has led to “overdetection”, i.e. an ability to clearly visualize even a small lesion sized a few millimeters, perform FNA, obtain a “suspicious” cytological conclusion and make a decision on surgical treatment; in fact, this may possibly be “overtreatment”. However, the postoperative histological conclusion “papillary thyroid microcarcinoma” is by no means “overdiagnosis” since it is a completely correct final pathological diagnosis fully corresponding to the current WHO Histological Classification of thyroid tumors (33).

Our study has certain strengths and limitations. Radiogenic and sporadic cases were selected from a single institution and were well matched by the place of residence. Although patients were not matched by age at surgery and sex, multivariate models were appropriately adjusted, and therefore confounding by these parameters is unlikely. Neither radiogenic, nor sporadic cases in the study were detected by active screening, implying that selection bias did not play a noticeable role. Some birth period or cohort effects might be expected stemming from the inclusion criterion for patients with sporadic MPTCs, which required that they were born since 1987 to ensure the lack of exposure to fallout. This led to the difference in calendar time of the first patient entry in the study between the radiogenic and sporadic groups (1992 and 2001, respectively; the proportion of patients with the radiogenic MPTCs diagnosed from 1992 to 2000 accounted for 18.4%). A rather limited number of patients in the pediatric and MPTCs measuring 1-5 mm subgroups might provide insufficient statistical power to detect the effects. Similarly, a very small number of recurrent tumors in the study and a relatively short follow-up period did not allow assessment of etiology-specific prognostic factors, pointing at the need for further research.

In conclusion, our study did not find evidence in support of important effects of internal irradiation on histopathological characteristics, more pronounced invasive properties and prognosis worsening for MPTCs in pediatric or young adult patients. These results imply that young MPTC patients with a radiation history (at least of internal exposure to radioiodine) may not require treatment strategies different from those for non-exposed MPTC patients. Of importance, some MPTCs in young patients may be locally advanced or develop distant metastasis indicating a need for risk stratification and tailored treatment, regardless of tumor etiology.

## Data availability statement

The original contributions presented in the study are included in the article. Further inquiries can be directed to the corresponding author.

## Ethics statement

The studies involving human participants were reviewed and approved by Bioethics Committee, State Institution V.P. Komisarenko Institute of Endocrinology and Metabolism of the National Academy of Medical Sciences of Ukraine, the Chornobyl Tissue Bank, Ethics Committee of Nagasaki University. Written informed consent to participate in this study was provided by the participants’ legal guardian/next of kin.

## Author contributions

TB, SC, LZ, NM, MT, SY, and VAS: study design and methodology. TB, SC, LZ, and MB: clinical and pathological data. LZ, TIR, and MI: investigation and formal analysis. SM: thyroid dosimetry. TB and VAS: statistical analysis, data interpretation, and writing of the manuscript. TB, SC, LZ, TIR, NM, MI, MT, MB, SM, SY, and VAS: revision of the manuscript. All authors have reviewed the manuscript and approved the final version.

## Funding

This research was supported in part by the Program of the Network-Type Joint Usage/Research Center for Radiation Disaster Medical Science, intramurally by the Atomic Bomb Disease Institute, Nagasaki University, and the Japan Society for the Promotion of Science (JSPS), KAKENHI Grant Numbers 19K07471, 19KK02670001, and 20KK0217.

## Acknowledgments

We gratefully acknowledge the commitment of the staff of the Laboratory of Morphology of Endocrine System and of the Department of Surgery of Endocrine Glands of IEM, who prepared all pathological material and operated on the patients, respectively. The authors gratefully acknowledge the confirmation of Ukrainian diagnoses by the International Pathology Panel of the Chornobyl Tissue Bank, which was supported by NCI grant number U24CA082102: A. Abrosimov, TB, G. Fadda, J. Hunt, MI, V. Livolsi, J. Rosai, E. D. Williams, N. Dvinskyh, and LZ.

## Conflict of interest

The authors declare that the research was conducted in the absence of any commercial or financial relationships that could be construed as a potential conflict of interest.

## Publisher’s note

All claims expressed in this article are solely those of the authors and do not necessarily represent those of their affiliated organizations, or those of the publisher, the editors and the reviewers. Any product that may be evaluated in this article, or claim that may be made by its manufacturer, is not guaranteed or endorsed by the publisher.
